# A Novel Combination of Wheat Peptides and Fucoidan Attenuates Ethanol-Induced Gastric Mucosal Damage through Anti-Oxidant, Anti-Inflammatory, and Pro-Survival Mechanisms

**DOI:** 10.3390/nu9090978

**Published:** 2017-09-06

**Authors:** Juntao Kan, Molly Hood, Charlie Burns, Jeff Scholten, Jennifer Chuang, Feng Tian, Xingchang Pan, Jun Du, Min Gui

**Affiliations:** 1 Nutrilite Health Institute, 720 Cailun Road, Shanghai 201203, China; junot.kan@Amway.com (J.K.); fred.tian@Amway.com (F.T.); 2 Nutrilite Health Institute, 7575 East Fulton Avenue, Ada, MI 49355, USA; molly.hood@Amway.com (M.H.); charlie.burns@Amway.com (C.B.); jeff.scholten@Amway.com (J.S.); 3 Nutrilite Health Institute, 5600 Beach Boulevard, Buena Park, CA 90621, USA; jennifer.chuang@Amway.com; 4 China National Research Institute of Food and Fermentation Industries, 24 Jiuxianqiao Middle Road, Beijing 100015, China; nutripan@163.com

**Keywords:** gastric mucosal damage, wheat peptides, fucoidan, anti-oxidant, anti-inflammation, pro-survival

## Abstract

Gastritis or peptic ulcer is believed to affect about half of people worldwide. Traditional medications can lead to adverse effects, therefore, alternative nutritional strategies are needed to prevent the development of gastric mucosal damage. A novel combination of two food-grade ingredients, wheat peptides and fucoidan (WPF), was prepared to treat male Sprague Dawley rats for 30 days before gastric mucosal damage was induced by oral administration of ethanol. The serum levels of biomarkers were determined by enzyme-linked immunosorbent assay. Biomarkers in stomach tissue were analyzed using immunohistochemistry. In addition, human gastric epithelial cell line (GES-1) was used to investigate protein expression by Western blot. WPF could attenuate ethanol-induced gastric mucosal damage in an inverse dose-dependent manner, with both ulcer index and pathological index improved. WPF increased superoxide dismutase level and decreased malondialdehyde level. WPF also decreased the levels of interleukin-8, platelet-activating factor, and Caspase 3, while increasing the levels of prostaglandin E-2, epidermal growth factor (EGF), and EGF receptor (EGFR). Furthermore, phosphorylation of EGFR and extracellular signal–regulated kinases was induced by WPF in GES-1 cells. In conclusion, the novel combination of wheat peptides and fucoidan attenuated ethanol-induced gastric mucosal damage in rats through anti-oxidant, anti-inflammatory, and pro-survival mechanisms.

## 1. Introduction

Gastric mucosal damage occurs when risk factors, such as alcohol, stress, *Helicobacter pylori*, and nonsteroidal anti-inflammatory drugs (NSAIDs), overwhelm protective factors/mechanisms, and this can lead to the development of gastritis, peptic ulcer, or even gastric cancer [1,2]. Particularly, immoderate alcohol consumption can cause mucosal edema, congestion, hemorrhage, epithelial exfoliation, and inflammatory cell infiltration, resulting in ulcers in the stomach [3,4]. Therefore, a rat model of ethanol-induced gastric mucosal damage is widely used to study preventive or protective effects of drugs or health foods [5,6].

Wheat peptides are biologically active peptides obtained by enzymatic hydrolysis from wheat protein [7,8], and they have anti-oxidant activity [9]. Wheat peptides could resist indomethacin-induced oxidative stress in intestinal epithelial cells-6 (IEC-6) and hydrogen peroxide-induced oxidative stress in pheochromocytoma cells-12 [10,11]. Furthermore, treatment with wheat peptides could protect against NSAID-induced small intestinal damage by decreasing oxidative stress in rats [12].

Fucoidan is a sulfated polysaccharide obtained mainly in various species of brown algae and brown seaweed such as *Undaria pinnatifida*, *Laminaria angustata*, *Fucus vesiculosus*, and *Fucus evanescens*, and it has anti-inflammatory activity [13,14]. Fucoidan can downregulate the production of interleukin-6 in colonic epithelial cells [15], and protect against aspirin-induced ulceration in rats through its immunomodulatory properties [16].

The protective mechanisms underlying ethanol-induced gastric mucosal damage mainly include anti-oxidation, anti-inflammation, and pro-survival [5,16,17]. Based on the anti-oxidant activity of wheat peptides and anti-inflammatory activity of fucoidan, we proposed a novel combination of wheat peptides and fucoidan (WPF), and tested its efficacy in a rat model of ethanol-induced gastric mucosal damage, as well as investigated possible involved mechanisms.

## 2. Materials and Methods

### 2.1. Materials and WPF Preparation

Wheat peptides were obtained from China National Research Institute of Food & Fermentation Industries (Beijing, China), and prepared by protease hydrolysis method [10]. The peptides included 3–6 amino acid residues, ranging from 140 to 1000 Da. Fucoidan (Beijing Gingko Group, Beijing, China) was extracted from *Laminaria japonica* (kelp) by water and filtered by membrane. An optimal ratio of 10:3 (wheat peptides:fucoidan) was determined after previous bioassays and in vivo pilot study as well as considering commercial cost and regulatory limits of China Food and Drug Administration (CFDA) on doses. WPF was prepared by Nutrilite Health Institute through mixture of 1 g of wheat peptides and 300 mg of fucoidan (daily intake for human).

### 2.2. Cell Culture

Human gastric epithelial cell line (GES-1) was obtained from the American Type Culture Collection (ATCC, Rockville, MD, USA) and was cultured at 37 °C under 5% CO_2_ in Dulbecco’s Modified Eagle Medium (Gibco, Grand Island, NY, USA) with 10% fetal bovine serum (Hyclone, Logan, UT, USA) and 1% penicillin streptomycin (Gibco, Grand Island, NY, USA).

### 2.3. Animals

Male Sprague Dawley rats (180–220 g) were purchased from Charles River (Beijing, China). Animals were housed in a temperature and humidity-controlled room (22–23 °C and 46–63%, respectively) and had free access to food and water. The experimental protocols (ethic code: SYXK (SU) 2013-0037) were approved by the Institutional Animal Care and Use Committee of Southeast University (Nanjing, China).

### 2.4. Ethanol-Induced Gastric Mucosal Damage Model

This model was established by following the protocol from CFDA for health food registration with the claim of “Assisting the Protection of Gastric Mucosa Function” [18]. After one-week acclimation, the animals were divided into eight groups (10 rats for each group): (1) naïve control; (2) model control; (3) low dose of WPF (108 mg/kg); (4) medium dose of WPF (217 mg/kg); (5) high dose of WPF (325 mg/kg); (6) fucoidan (50 mg/kg); (7) wheat peptide (167 mg/kg); (8) cimetidine (65 mg/kg). The rats of model control and treatment groups were given either vehicle (saline) or test articles by daily oral gavage for 30 days.

Acute model of gastric mucosal damage was induced by oral administration of ethanol (1 mL) 24 h after the 30-day-treatment period. Rats were sacrificed 1 h after ethanol treatment. The naïve control group received vehicle only. Prior to termination, blood was collected and centrifugated to get serum for further analysis. Then the animals were euthanized by CO_2_ inhalation, and stomach was excised for macroscopic and histopathologic evaluation.

### 2.5. Macroscopic Analysis

The excised stomach was opened along the greater curvature, and mucosa was washed with cold phosphate-buffered saline (PBS). Gastric mucosal damage was scored by an experienced gastroenterologist, who was blinded to the samples, and expressed as ulcer index (Table 1, [18]).

### 2.6. Histopathologic Evaluation

The stomach tissue was then fixed in 10% formaldehyde, embedded into paraffin, and cut into 4 μm sections. A commercially available hematoxylin-eosin (H&E) kit (Beyotime, Shanghai, China) was used to stain sections. An expert pathologist evaluated the slides in a blinded fashion and calculated the total pathological index as follows (Table 2, [18]).

### 2.7. Immunohistochemistry

Caspase 3 and epidermal growth factor receptor (EGFR) in paraffin sections were visualized with rabbit polyclonal Caspase 3 antibody (Abcam, Cambridge, MA, USA) and EGFR antibody (Zen BioScience, Chengdu, China) followed by staining with a horseradish peroxidase (HRP)-conjugated secondary antibody, and diaminobenzidine substrate (Maixin, Fuzhou, China). Five random fields from each section were photographed with a Zeiss digital camera, and analyzed using a semi-quantitative scoring system as follows (Table 3, [19,20]).

### 2.8. Biochemical Analysis

Serum levels of malondialdehyde (MDA), superoxide dismutase (SOD), platelet-activating factor (PAF), prostaglandin E-2 (PGE2), epidermal growth factor (EGF), and interleukin-8 (IL-8) were determined by using commercially available kits (JRdun Biotechnology, Shanghai, China) according to the manufacturer’s instructions.

In brief, the MDA assay kit uses thiobarbituric acid (TBA) to react with MDA to generate a MDA-TBA adduct which can be easily quantified colorimetrically at 532 nm. The SOD assay kit uses an enzyme mix that oxidizes WST-1 (2-(4-iodophenyl)-3-(4-nitrophenyl)-5-(2,4-disulfophenyl)-2H-tetrazolium, monosodium salt) and produces a water-soluble formazan dye to generate color that is read in a plate reader at 440 nm.

The levels of PAF, PGE2, EGF, and IL-8 were determined by sandwich enzyme-linked immunosorbent assay (ELISA). The principle of the assay is to capture each molecule from samples in the wells of a microtiter plate coated by a pre-titered amount of primary antibody and then the binding of biotinylated polyclonal antibody to the captured molecule. Next, horseradish peroxidase is added to wells to bind to the immobilized biotinylated antibody, and quantification of immobilized antibody-enzyme conjugate is determined by horseradish peroxidase activities in the presence of the substrate 3,3′,5,5′-tetramethylbenzidine. The enzyme activity is measured spectrophotometrically by the increased absorbency at 450 nm, corrected from the absorbency at 590 nm, after acidification of formed product.

### 2.9. Western Blot

GES-1 cells were treated with control (PBS) or WPF (1.3 mg/mL) for 12 h before protein extraction with cell lysis buffer (Cell Signaling, Danvers, MA, USA). Lysates were boiled with 5× loading buffer and then loaded onto sodium dodecyl sulfate-polyacrylamide gels. The separated proteins were transferred to polyvinylidene fluoride (PVDF) membranes (Millipore, Billerica, MA, USA), then blocked with 5% nonfat dry milk in Tris-buffered saline/Tween (TBST). Primary and secondary antibodies were used to successively incubate the PVDF membrane. The HRP-conjugated protein was detected by chemiluminescent horseradish peroxidase substrate solution (Millipore, Billerica, MA, USA). The primary antibodies against EGFR, p-EGFR, extracellular signal-regulated kinases (ERK), and p-ERK were purchased from Cell Signaling, and GAPDH antibody was purchased from Abcam.

### 2.10. Statistical Analysis

Statistical analysis was performed using SPSS 13.0 software (SPSS Inc., Chicago, IL, USA). Quantitative data are presented as mean ± standard deviation (SD). Differences between groups were analyzed using Student’s *t*-test when only two groups were compared or assessed by one-way analysis of variance (ANOVA) with Dunnett’s *post-hoc* test when more than two groups were compared. A significant effect was defined as *p* < 0.05.

## 3. Results

### 3.1. WPF Attenuated Ethanol-Induced Gastric Mucosal Damage

**Figure 1:**
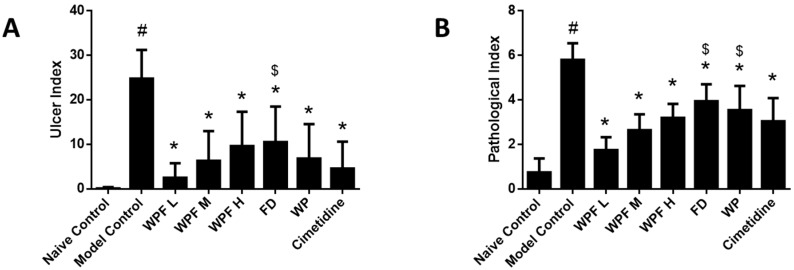
Wheat peptides and fucoidan (WPF) attenuated ethanol-induced gastric mucosal damage. The rats were treated with different doses of WPF (108 mg/kg, 217 mg/kg, and 325 mg/kg), fucoidan (50 mg/kg), wheat peptide (167 mg/kg), or cimetidine (65 mg/kg) for 30 days before oral administration of ethanol to induce gastric mucosal damage. The stomach was excised 1 h after ethanol treatment for macroscopic and histopathologic evaluation. (**A**) Ulcer index; (**B**) Pathological index. Data are presented as mean ± SD. *N* = 10. ^#^ *p* < 0.05 vs. naïve control, * *p* < 0.05 vs. model control, ^$^ *p* < 0.05 vs. low dose of WPF. WPF L: Low dose of WPF; WPF M: Medium dose of WPF; WPF H: High dose of WPF; FD: Fucoidan; WP: Wheat peptides.

Oral administration of ethanol induced gastric mucosal damage, resulting in an ulcer index from (0.1 ± 0.3) to (24.8 ± 6.5) (*p* < 0.05, Figure 1A). WPF attenuated ethanol-induced mucosal damage, with ulcer indexes of 2.5 ± 3.3, 6.3 ± 6.7, 9.6 ± 7.7, respectively (*p* < 0.05 vs. model control). The low dose of WPF showed better protective effects when compared with the treatment of fucoidan alone (*p* < 0.05 vs. fucoidan).

**Figure 2:**
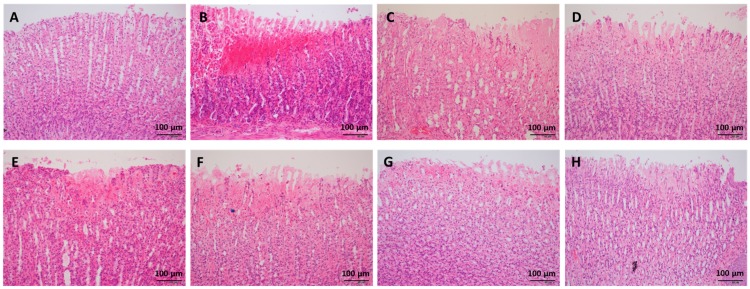
Representative photos of histopathologic evaluation. The stomach tissue was fixed in 10% formaldehyde, embedded into paraffin, and cut into 4 μm sections for hematoxylin-eosin (H&E) staining. (**A**) Naïve control; (**B**) Model control; (**C**) Low dose of WPF (108 mg/kg); (**D**) Medium dose of WPF (217 mg/kg); (**E**) High dose of WPF (325 mg/kg); (**F**) Fucoidan (50 mg/kg); (**G**) Wheat peptide (167 mg/kg); (**H**) Cimetidine (65 mg/kg).

H&E staining indicated that ethanol administration led to severe congestion and hemorrhagic erosions in the stomach tissue, as well as degeneration and necrosis of the mucosal epithelial cells (Figure 2). Low, medium, and high doses of WPF significantly decreased the pathological index from (5.8 ± 0.8) to (1.8 ± 0.6) and from (2.7 ± 0.7) to (3.2 ± 0.6), respectively (*p* < 0.05, Figure 1B). The low dose of WPF showed better protective effects then fucoidan or wheat peptides alone (*p* < 0.05 vs. fucoidan, *p* < 0.05 vs. wheat peptides). The histopathological data were consistent with the macroscopic results (ulcer index).

### 3.2. Anti-Oxidant Effect of WPF

**Figure 3:**
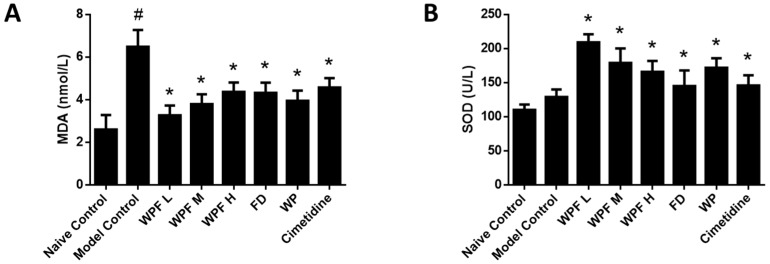
Anti-oxidant effect of WPF. Serum levels of malondialdehyde (MDA) (**A**) and superoxide dismutase (SOD) (**B**) were determined by using commercially available colorimetric kits. Data are presented as mean ± SD. *N* = 10. ^#^ *p* < 0.05 vs. naïve control, * *p* < 0.05 vs. model control. WPF L: Low dose of WPF; WPF M: Medium dose of WPF; WPF H: High dose of WPF; FD: Fucoidan; WP: Wheat peptides.

In the model group, the level of MDA, a marker for oxidative stress, was significantly increased (*p* < 0.05 vs. naïve control, Figure 3A), suggesting high oxidative stress was induced during gastric mucosal damage. Treatment with WPF could maintain MDA to a normal level (*p* < 0.05 vs. model control). Likewise, WPF increased the level of SOD, an antioxidant enzyme (*p* < 0.05 vs. model control, Figure 3B).

### 3.3. Anti-Inflammatory Effect of WPF

**Figure 4:**
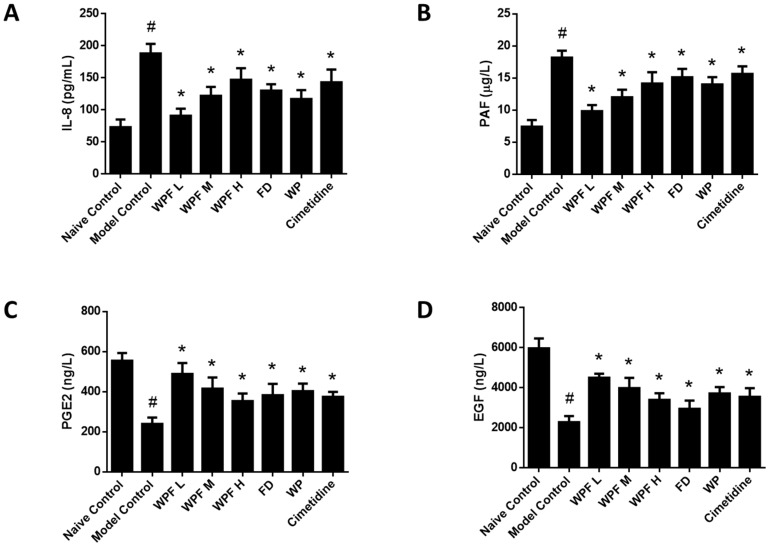
Anti-inflammation and pro-survival effects of WPF. Serum levels of interleukin (IL)-8 (**A**), platelet-activating factor (PAF) (**B**), prostaglandin E-2 (PGE2) (**C**), and epidermal growth factor (EGF) (**D**) were determined by using commercially available enzyme-linked immunosorbent assay (ELISA) kits. Data are presented as mean ± SD. *N* = 10. ^#^ *p* < 0.05 vs. naïve control; * *p* < 0.05 vs. model control. WPF L: Low dose of WPF; WPF M: Medium dose of WPF; WPF H: High dose of WPF; FD: Fucoidan; WP: Wheat peptides.

Ethanol administration significantly induced the secretion of IL-8, which could be reversed by treatment with WPF (*p* < 0.05, Figure 4A). As a mediator of allergic and inflammatory processes, PAF was also inhibited by treatment with WPF (*p* < 0.05, Figure 4B).

### 3.4. Pro-Survival Effect of WPF

**Figure 5:**
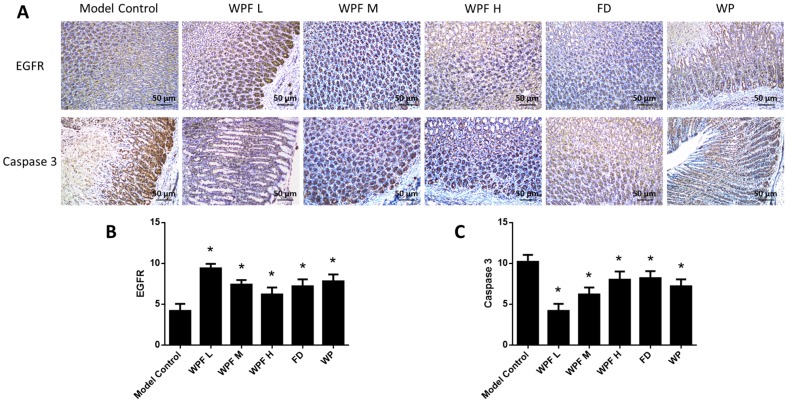
Pro-survival effect of the WPF. (**A**) Biomarkers in stomach tissue were stained using immunohistochemistry. Epidermal growth factor receptor (EGFR) (**B**) and Caspase 3 (**C**) were analyzed with a semi-quantitative scoring system. Data are presented as mean ± SD. *N* = 10. * *p* < 0.05 vs. model control. WPF L: Low dose of WPF; WPF M: Medium dose of WPF; WPF H: High dosage of WPF; FD: Fucoidan; WP: Wheat peptides.

The secretion of PGE2 and EGF, main gastroprotective factors, were highly inhibited under the gastric mucosal damage model, while WPF could induce PGE2 and EGF secretion to promote cell survival (*p* < 0.05, Figure 4C,D). Furthermore, we investigated the stomach tissue by immunohistochemistry semi-quantitative analysis to reveal that expression of EGFR was also induced by WPF treatment (*p* < 0.05, Figure 5A,B), while the expression of Caspase 3, an apoptosis activator, was reduced (*p* < 0.05, Figure 5A,C), suggesting a pro-survival effect of the formula.

### 3.5. WPF-Induced Activation of EGFR-ERK Pathway

**Figure 6:**
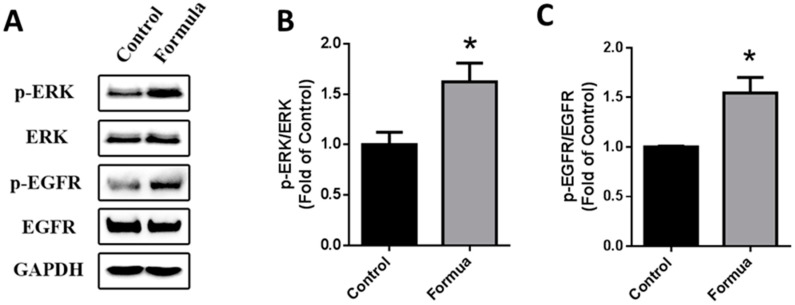
WPF-induced activation of EGFR-ERK pathway. Human gastric epithelial cell line (GES-1) cells were treated with control (PBS) or WPF (1.3 mg/mL) for 12 h before protein was extracted by cell lysis buffer and analyzed using Western blot. GAPDH was used as a loading control. (**A**) Representative blots of p-ERK, ERK, p-EGFR and EGFR; (**B**) Changes of p-ERK/ERK; (**C**) Changes of p-EGFR/EGFR. Data are presented as mean ± SD. * *p* < 0.05 vs. control. Experiments repeated at least three times.

Next, we treated GES-1 cells with WPF to investigate possible molecular mechanisms. Protein expression of EGFR and ERK was not changed by WPF, while phosphorylation of EGFR and ERK was significantly induced by WPF (*p* < 0.05, Figure 6), showing that the EGFR-ERK pathway could be involved in the pro-survival effect of the WPF.

## 4. Discussion

Gastritis or peptic ulcer is believed to affect about half of people worldwide, and traditionally they are treated by medications such as antacids, histamine H_2_ receptor antagonists, or proton pump inhibitors [21]. However, the adverse effects of these therapies are known, including hypersensitivity, arrhythmia, impotence, gynecomastia, and hematopoietic changes [22]. Bioactive ingredients extracted from food resources can provide alternative nutritional strategies without intolerable side effects to prevent the development of gastric mucosal damage into gastritis or peptic ulcer [23,24,25]. In this study, we showed that a novel combination of two food-grade ingredients, wheat peptides and fucoidan, could attenuate ethanol-induced gastric mucosal damage, with both macroscopic and histopathologic evaluation confirmed.

Three doses of WPF were used in the ethanol-induced gastric mucosa damage model to assess an optimal dose. In our current study, the lowest treatment dose provided the most benefit by maintaining values for ulcer index, pathological index, MDA, SOD, IL-8, PAF, PGE2, and EGF near naïve control. The higher doses of WPF tested also provided statistically significant protection from mucosal damage, but to a lesser extent. This inverse dose response has been reported previously [12,26,27], however, more work needs to be done to determine if the lowest dose tested is the optimal dose, and what would be the minimally effective dose. This study did establish that the combination of wheat peptides and fucoidan is effective in protecting the gastric mucosa from ethanol-induced damage.

The pathogenesis of ethanol-induced gastric mucosal damage is usually complex [3,4], in which oxidative stress and depletion of antioxidants have been considered as a critical step in ethanol-induced mucosal damage [5,17]. MDA is the principal and most studied product of polyunsaturated fatty acid peroxidation, and is a biomarker for oxidative stress [28,29]. SOD has been identified as an important antioxidant defense in nearly all living cells exposed to oxygen [30]. In the ethanol-induced gastric mucosal damage model, the level of MDA was increased while the level of SOD was decreased [31,32]. We also found the similar trend in our model and WPF could maintain the antioxidant defense.

Inflammation is another critical mechanism in ethanol-induced gastric mucosal damage [16,33], in which pro-inflammatory cytokines, such as IL-1, IL-6, and IL-12, are elevated, while anti-inflammatory cytokines, such as IL-4 and IL-10, are decreased [16,34,35]. We first reported IL-8 secretion was increased under gastric mucosal damage, which could be attenuated by WPF. PAF is an endogenous phospholipid which has been implicated as a mediator of allergic and inflammatory processes and most potent gastric ulcerogen [36,37]. It is synthesized and released by inflammatory cells including neutrophils [36], which was found to infiltrate in the injured gastric mucosa [38,39]. The inhibitory effect on PAF indicated that WPF can mediate inflammatory processes.

PGE2 and EGF exhibit gastroprotective and ulcer healing properties, mainly due to their mitogenic, pro-survival, and anti-apoptotic actions [40,41,42]. In addition, EGF could increase gastric blood flow to protect mucosa [43,44]. In the present model, the level of PGE2 and EGF was reduced by ethanol, which was consistent with previous studies [45,46]. Furthermore, EGF and PGE2 could upregulate Bcl-2 and prevent Cytochrome C release from mitochondria to activate Caspase 3 [42]. In our study, we also found decreased expression of Caspase 3 in stomach tissue accompanied with an elevated level of PGE2 and EGF in serum after treatment with WPF.

EGFR, a transmembrane receptor tyrosine kinase, is very critical in wound healing [47]. High expression of EGF/EGFR in damaged gastric mucosa may be important for the repair of gastric mucosa [48]. In our study, the expression of EGFR in stomach tissue was inversely related to the extent of gastric mucosal damage. In a previous report, inhibition of tyrosine kinase activity of EGFR attenuated gastric mucosal regeneration [49]. Here, we showed that WPF could induce the phosphorylation of EGFR to protect gastric epithelial cells, in addition to the phosphorylation of ERK, an intracellular signaling molecule downstream of EGFR [50,51], suggesting that the EGFR-ERK pathway was involved in a pro-survival effect of WPF.

## 5. Conclusions

Taken together, we have shown the protective effects of a novel combination of wheat peptides and fucoidan in a rat model of ethanol-induced gastric mucosal damage, and disclosed anti-oxidant, anti-inflammatory, and pro-survival mechanisms of WPF. More importantly, these findings shed new light on alternative nutritional strategies without intolerable side effects to prevent the development of gastric mucosal damage.

## Figures and Tables

**Table 1:** Scoring criteria for ulcer index.

Grade	1	2	3	4
Spot Erosion	Each spot			
Linear Erosion (Length)	1–5 mm	5–10 mm	10–15 mm	>15 mm
Linear Erosion (Width)	1–2 mm	>2 mm		
Ulcer Index = Spot Erosion + Linear Erosion Length + (Linear Erosion Width) × 2				

**Table 2:** Scoring criteria for pathological index.

Grade	1	2	3	4	5
Congestion	<20%	20–40%	40–60%	60–80%	≥80%
Hemorrhage	<20%	20–40%	40–60%	60–80%	≥80%
Necrosis	<20%	20–40%	40–60%	60–80%	≥80%
Pathological Index = Congestion + Hemorrhage × 2 + Necrosis × 3					

**Table 3:** Semi-quantitative scoring system.

Grade	0	1	2	3	4
Percentage of Positive Cells	≤5%	6–25%	26–50%	51–75%	>75%
Intensity of Immunostaining	No	Low	Moderate	Strong	
Total Score = Percentage of Positive Cells × Intensity of Immunostaining					
